# Vision-Based Multirotor Following Using Synthetic Learning Techniques

**DOI:** 10.3390/s19214794

**Published:** 2019-11-04

**Authors:** Alejandro Rodriguez-Ramos, Adrian Alvarez-Fernandez, Hriday Bavle, Pascual Campoy, Jonathan P. How

**Affiliations:** 1 Computer Vision and Aerial Robotics group, Centre for Automation and Robotics, Universidad Politécnica de Madrid (UPM-CSIC), Calle Jose Gutierrez Abascal 2, 28006 Madrid, Spain; hriday.bavle@upm.es (H.B.); pascual.campoy@upm.es (P.C.); 2 Artificial Intelligence group, University of Groningen, 9712 Groningen, The Netherlands; a.alvarez.fernandez@student.rug.nl; 3 Aerospace Controls Laboratory, Massachusetts Institute of Technology (MIT), 77Mass. Ave., Cambridge, MA 02139, USA; jhow@mit.edu

**Keywords:** multirotor, UAV, following, synthetic learning, reinforcement learning, deep learning

## Abstract

Deep- and reinforcement-learning techniques have increasingly required large sets of real data to achieve stable convergence and generalization, in the context of image-recognition, object-detection or motion-control strategies. On this subject, the research community lacks robust approaches to overcome unavailable real-world extensive data by means of realistic synthetic-information and domain-adaptation techniques. In this work, synthetic-learning strategies have been used for the vision-based autonomous following of a noncooperative multirotor. The complete maneuver was learned with synthetic images and high-dimensional low-level continuous robot states, with deep- and reinforcement-learning techniques for object detection and motion control, respectively. A novel motion-control strategy for object following is introduced where the camera gimbal movement is coupled with the multirotor motion during the multirotor following. Results confirm that our present framework can be used to deploy a vision-based task in real flight using synthetic data. It was extensively validated in both simulated and real-flight scenarios, providing proper results (following a multirotor up to 1.3 m/s in simulation and 0.3 m/s in real flights).

## 1. Introduction

Currently, learning techniques aided by synthetic information are increasingly popular in the robotics field, mostly due to the lack of available real-domain data information for application-specific tasks, for example, low-level motion control for complex robot dynamics [1,2,3] or vision-based strategies for unusual scenarios [4,5,6]. In addition, with the rise of deep learning [7] and multiple-layer models, the amount of data required for the stability of learning and model generalization has grown several orders of magnitude, which makes dataset generation harder. State-of-the-art reinforcement-learning techniques are also still notably sample-inefficient [8], which leads to needing a high number of agent–environment interactions, which is normally not possible in the real world due to system stability, sample randomness or power availability. In this context, in order to achieve scalability for both deep- and reinforcement-learning techniques, the exploration of synthetic-learning strategies in robotics is a matter of importance that is not yet resolved. This paradigm is potentially valuable when annotated datasets are not available and/or there is highly nonlinear mapping between several heterogeneous information sources, for example, navigation-throughout-cluttered indoor and outdoor environments using simulated image cues [9].

In the context of synthetic-data generation by robotic simulators, there is an explicit differentiation between robot-dynamics simulation and photorealistic-image generation and they are normally treated separately. Realistic simulation of robot dynamics is important in the field of imitation- or reinforcement-learning algorithms, that is, more realistic models and/or physics can lead to more effective performance in the real world. In this regard, novel system dynamics can be realistically simulated to be controlled with learning-based (or classical) approaches and without the necessity of building the actual robot. Although there are inconsistencies between virtual and real models, differences between simulated and real systems can be tackled with several methods (e.g., References [1,2,3]). Within the scope of the generation of photorealistic images, there is still a clear ceiling in the achieved realism for current simulators. Light, polygons and material simulations have intrinsic limitations that can be very evident for a human observer. Nevertheless, they can be extremely useful in the field of vision-based strategies, such as object detectors, along with other transfer-learning or domain-adaptation techniques (e.g., References [10,11,12]).

In this scenario, a multirotor can be a suitable robotic test bench to validate this type of synthetic technique for robotic applications. Their inherent open-loop system instability, which can create a stressful scenario for the technique under research and their low-cost RGB camera, which can lead to a wide variety of applications, make this platform a suitable target for novel motion-control research, as well as for vision-based strategies. In this respect, the versatility of this platform and the lack of complete learning-based techniques aided by synthetic data compose the base motivation for the studied application case in this work. Indeed, the multirotor following task introduced in this study was proposed as a baseline task due to its challenging nature and increasing interest of the research community (e.g., International robotics competition: https://www.mbzirc.com/). At the same time, since multirotors have become the center of attention of many industrial automation processes, the capacity of multirotor detection with a low-cost sensor and the ability to follow a potential noncooperative multirotor is an interesting topic of research.

In particular, this paper explores an scenario where a noncooperative target multirotor has the ability to move in the 3-Degrees of Freedom (DOF) of space and the follower multirotor, which implemented our approach, specifically relies on its low-level attitude Flight Controller (FC) and its monocular RGB camera to accomplish the complete following maneuver. We focused on a novel object-following case where the follower RGB camera was also able to move in two directions of space (camera gimbal pan and tilt), providing higher dimensionality and versatility to the approach. The entire behavior was learned with synthetic data by means of two strategies based on deep and reinforcement learning for multirotor detection and motion control, respectively.

## 2. Contribution

**Figure 1:**
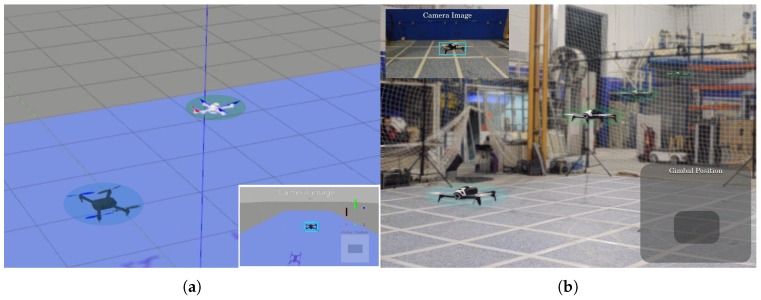
Image composition of a noncooperative multirotor (light-blue) followed by another multirotor that implemented our synthetic-learning-based approach (light-green) in the (**a**) simulated and (**b**) real experimentation environment.

In this study, monocular vision-based following of a noncooperative multirotor using synthetic-learning techniques is presented. The aim was to establish a complex real-world multirotor application based on synthetic data, by means of deep- and reinforcement-learning methods. The main contributions of this work are listed below:

- A robust detection technique was trained with a novel approach based on synthetic photorealistic images generated by a commercial game engine. The synthetic multirotor image dataset, utilized in this work for detector training, was also released as an open-source dataset.
- Problem formulation under the reinforcement-learning framework was designed in order to achieve learning convergence for a motion-control agent with a state-of-the-art deep reinforcement-learning algorithm and within the context of high-dimensional continuous state and action spaces. The agent was trained in a simulated environment and tested in real-flight experiments (refer to Figure 1).
- A novel motion-control strategy for object following is introduced where camera gimbal movement is coupled with multirotor motion during multirotor following.

Additionally, real-flight tests were performed in a broad variety of conditions in order to validate the virtual-to-real approach and with low-cost on-board sensors as single raw data inputs. Results prove that our present framework can be used for vision-based tasks in a real flight using synthetic data. The remainder of the paper is organized as follows: Section 4 describes our approach and provides a detailed explanation about the multirotor detector and the motion-control policy, explaining the problem formulation and the system architecture. Section 5 outlines the carried-out experiments and their corresponding results and Section 6 remarks on and discusses the most relevant experimentation outcomes. Finally, Section 7 concludes the paper and indicates future lines of research.

## 3. Related Work

In this paper, the task and techniques under study were approached with strategies of diverse nature and, due to this, there are several related works that are adjacent to the case under study. In the following section, the key aspects of the most relevant related works are described.

### 3.1. Autonomous Aerial Pursuit

Autonomous aerial pursuit, that is, Unmanned Aerial Vehicle (UAV)-to-UAV pursuit, has been an underlying field of research during the last decade. This line of research has not been the focus of UAV research but has provided theoretical contributions mostly validated on numerical simulations. Hence, most innovations assumed ideal data information sources and were tested only by means of numerical simulations [13,14,15,16,17].

In References [14,15,16], the target UAV state was assumed to be known and considered cooperative under the case of study. In Reference [17], a sliding-mode approach with proper Lyapunov function was explored and validated on numerical simulations. In Reference [16], both pursuit algorithms and vision-based theoretical strategies were explored under the assumption of ideal sensors and validated on numerical simulations. A cooperation strategy for multiple UAVs to pursue a target moving in an adversarial environment where threat exposure should be minimized was studied in Reference [13]. A probabilistic model of the environment was provided and the information exchange between the cooperative UAVs is assumed to be available in a numerical simulation.

### 3.2. Monocular Vision-Based Object Following

Several works researched the topic of vision-based autonomous object following with UAVs. Most of them explored classical control schemes (e.g., Proportional Integral Derivative (PID) control [18]) with classical computer-vision techniques (e.g., color-based detectors [19]). In Reference [20], a nonlinear adaptive observer was used for target-object estimation and a guidance law for target-object tracking was developed. In this case, the vision sensor was modeled and the complete approach was validated under numerical simulations.

Some approaches rely on an online user-defined Region-of-Interest (RoI) within the image plane during the execution of a real flight in order for the UAV to be able to follow a tracked object [18,21,22]. In Reference [18], a PID control scheme was used to control a multirotor aerial robot based on the OpenTLD [23] tracker in the image plane. In Reference [21], the authors developed a novel frequency- and image-plane-domain tracker with latter pose estimation of the object and standard PID control. In Reference [22], a state-of-the-art tracker [24] was used and a standard PID control scheme within the image plane was established. The stated work also performed real-flight validation tests and developed a novel camera handover strategy in order to enable long-term operation through several UAVs. As other close related works, some strategies use learning techniques for orientation tracking with respect to image patches based on state-of-the-art detectors and trackers [25,26].

Furthermore, some techniques carried out specific-object detection in conjunction with continuous-tracking strategies, such as Kalman or particle filters [19,27,28]. In References [27,28], a strategy for human following was designed and tested in real flights. In Reference [27], a miniature robotic blimp was used as the robotic platform, with human-pose estimation and standard PID control. In Reference [28], human tracking was carried out over a long period of time and long distances by means of active infrared markers and the estimation of the pose of both human and UAVs without calibrated or stationary cameras. In Reference [19], a color-based detector fed a particle filter that aided a pose-based PID control scheme.

Finally, some other strategies aimed at separately controlling a camera gimbal for object tracking, in which the detected object is kept centered in the image plane over time [29,30]. In Reference [29], a ground-based camera gimbal is able to track a remote object. In Reference [30], a camera gimbal was mounted as payload on a fixed-wing UAV for target tracking during the execution of the test flights.

### 3.3. Reinforcement Learning for Real-World Robotics

Several state-of-the-art techniques aim at training reinforcement-learning agents with synthetic data but by targeting their deployment in the real world. Some techniques focus on learning high-dimensional system dynamics for motion control [1,2,3]. The authors of Reference [1] utilized precise simulations and randomized system dynamics for quadruped locomotion learning with continuous states and actions. In Reference [2], high-level velocity control was learned with ground-truth simulations in order to develop a UAV task-specific landing maneuver on top of a moving platform. Validation tests were carried out through a motion-capture system in the real world. In Reference [3], the dynamics of a multirotor UAV were precisely simulated in order to train a reinforcement-learning agent for low-level attitude control. The learned policies were directly validated in the real world with a motion-capture system.

On the other hand, some strategies intend to overcome the virtual-to-reality gap for vision-based applications [9,31,32,33,34,35]. In Reference [9], synthetic-data adaptation was performed using an application-dependent Cycada pipeline for ground-robot navigation. In Reference [31], domain-randomization techniques were used to train a deep reinforcement-learning algorithm for dexterous in-hand manipulation. The authors of Reference [32] randomized simulated images to generate a canonical environment for hand manipulation.

Moreover, multirotor-related vision-based applications have also been studied [33,34,35,36]. In Reference [33], a color-based detector and an image-based deep reinforcement-learning policy controller were utilized for object following with an off-the-shelf multirotor. In Reference [36], human commands were mixed with reinforcement-learning exploration–exploitation actions in order to improve safety for multirotor navigation. The authors of Reference [35] used the Monte Carlo policy-evaluation method for vision-based navigation learning with synthetic images. In Reference [34], small-size multirotor navigation was achieved through a combination of real-world data to learn about robot dynamics and synthetic data for vision-based collision avoidance.

### 3.4. Multirotor In-Flight Detection and Tracking

Multirotor in-flight detection and tracking is increasingly becoming a topic of interest for the research community since the successful application of multirotor UAVs in several automation processes in the industry. Most strategies rely on motion detection during flight for a relatively reduced subset of UAV types [10,11,12,37,38]. In Reference [10], Convolutional Neural Networks (CNNs) were used for camera-motion estimation and temporal information was exploited to detect UAVs and aircrafts. In Reference [11], pixels corresponding to the background in the image were estimated and a Kalman filter was utilized to track motion of a UAV. In Reference [12], UAV detection and tracking required the exchange of information between both UAVs. The authors of Reference [37] explored Haar-like features, Histogram of Gradients (HOG) and Local Binary Patterns (LBP) using cascades of boosted classifiers for outdoor UAV detection and tracking. In Reference [38], extensive flight experimentation was carried out with real-time performance for general object tracking.

### 3.5. Object Detection with Synthetic Images

Deep learning is de facto the most used technique for object detection. Nevertheless, it usually requires a large amount of domain-specific data to achieve generalization. In this context, the research community is exploring the incorporation of synthetic images into the training process due to their availability and capabilities [4,5,6,39,40,41,42]. In Reference [4], synthetic images were optimized to match object-detector-extracted features. These images were used for real-image dataset augmentation [4]. The authors of Reference [5] randomized simulated images for 3D object localization; objects were composed of low-dimensional feature textures and shapes.

In Reference [40], synthetic images were generated by adding background and textures to simple 3D Computer-Aided Design (CAD) models. Features were extracted with a CNN trained on ImageNet and a classifier was trained for the final stage. In Reference [6], adversarial training was used to train Faster R-CNN [43] for cross-domain adaptation. The domains were adapted at the image level (e.g., image style and illumination) and instance-level representation (e.g., object appearance and size shift). In Reference [39], simple synthetic objects were detected through backbone-network (feature extractor) weight freezing and fine-tuning the last layers. Validation tests were performed with Faster R-CNN, R-FCN [44] and Mask R-CNN [45]. In Reference [41], adversarial training was used for simulated-to-real domain transferring. The dataset was generated through synthetic-image refinement and automated labeling. Finally, novel approaches showed outstanding performance when trained with small real-world datasets, outperforming previous pretrained architectures [46], such as SSD or Faster R-CNN. These strategies are potentially usable in the field of synthetic-dataset integration .

## 4. Vision-Based Multirotor Following Approach

In this paper, the vision-based autonomous following of a Noncooperative Multirotor (NC-M) is presented. Our approach is mainly composed of a multirotor detector, an image-based tracker and motion-control policy.

### 4.1. Multirotor Detector

**Figure 2:**
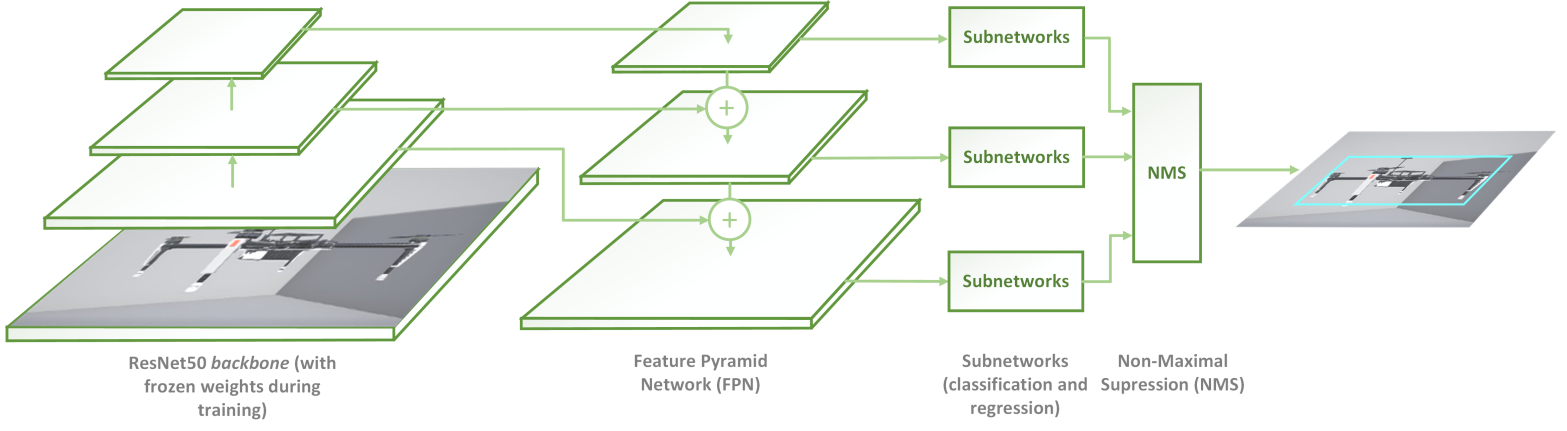
Architecture of proposed multirotor detector, based on RetinaNet architecture, with ResNet50 backbone and five levels for Feature Pyramid Network (FPN; fewer levels represented in figure for the sake of simplicity). Backbone weights were frozen during training. Final subnetworks were fine-tuned with pure synthetic multirotor images.

A deep-learning-based detector was trained by means of synthetic multirotor images with a simple background (see Section 5.2.1) and with the aim of extracting an accurate RoI of the NC-M. The detector is able to generalize enough from synthetic multirotor images and provide state-of-the-art accuracy and inference time. The detector used in this study was a custom implementation of the one-stage RetinaNet detector [47], which is a single unified network composed of a backbone network and two task-specific subnetworks (refer to Figure 2). The backbone is responsible for computing a convolutional and pyramidal feature map over an entire input and is an off-the-shelf convolutional network. In this work, ResNet50 was selected as the full network backbone in order to find a balanced trade-off between accuracy and image throughput [48].

The key aspects for RetinaNet to be suitable for learning new pure synthetic classes are its rich feature maps, generated by its Feature Pyramid Network (FPN) architecture and its Focal Loss (FL) design, which is a specific case of a weighted cross-entropy loss function. RetinaNet was also proven to be the best architecture in terms of accuracy and speed, compared with Faster R-CNN, R-FCN and other state-of-the-art architectures, in the wide variety of conditions presented in Reference [47]. Our approach incorporated five-layer levels from the backbone for FPN feature-map generation. FL minimizes the effect of large class imbalance (diminishing the influence of already well-learned classes) and encourages learning those samples that have not yet been properly learned (i.e., particular losses of already learned samples can overwhelm whole loss computation), as shown in Equations (1) and (2).

(1) $$FL\left(p_{t}\right)=-\alpha_{t}{\left(1-p_{t}\right)}^{\eta }log\left(p_{t}\right)$$

(2) $$p_{t}=\left\{\begin{array}{cc} p & \;\mathrm{if}\;y=1\\ 1-p & \;\mathrm{otherwise} \end{array}\right.,$$

where η is the focusing parameter and $\alpha_{t}$ is the classical α−balanced loss parameter [47]. This model architecture enables the detection (RoI) of a real NC-M within the image plane, which can be used by the next stages of the approach.

### 4.2. Image-Based Tracker

The stated multirotor detector can infer the RoI of the detected NC-M. Nevertheless, due to the multirotor perspective, illumination changes or glitches in the video feed, the multirotor detector could fail to regress a RoI, even when the NC-M is present in the image and the detector is able to run in real time. In this scenario, in order to provide a continuous RoI of the NC-M and to improve overall performance, our approach incorporated an adapted implementation of the state-of-the-art ECO tracker [49] that can track the RoI of the NC-M in every camera frame during the following maneuver.

The key aspects of the ECO tracker that make it suitable for these dynamic-feature frames are its robust hand-crafted features for tracking and its factorized convolution design. The hand-crafted features are computationally efficient and robust. The factorized convolution operator dramatically reduces the number of parameters of the Discriminative Correlation Filter (DCF), allowing for real-time tracking (>20 Hz) on an average CPU. In this context, the tracker is able to provide a RoI at every time step. However, when the detector provides a new RoI (with a predefined threshold of uncertainty that assures robustness), the current tracker RoI is updated for further tracking.

### 4.3. Motion-Control Policy

Deep reinforcement-learning techniques were applied for learning the multirotor motion-control policy for object following. The complete learned behavior is challenging due to the coupled nature of actuation–response of the system, that is, the multirotor system was coupled with the camera gimbal actuation since different trajectories of actuation (in both the multirotor and the camera gimbal) could lead to the same result in the image plane. This characteristic of the system is further explored in Section 5.

Considering the reinforcement-learning framework, an agent and an environment compose the two main elements that are defined to interact with each other. The stated interaction is carried out through agent actions that occur in an instantaneous effect in the environment, (returning the effect consequence back to the agent, that is, agent state) and through environment rewards that guide the evolution of the agent behavior. Thus, in the reinforcement-learning paradigm, an agent is determined to generate a policy $\pi (s_{t})$ in order to maximize its expected accumulated reward $R_{t}$ through the execution of an action $a_{t}\in A$ for each state $s_{t}\in S$ at any step in time *t*. The expected return, given a state and an action in time *t*, is

(3) $$Q^{\pi }\left(s_{t},a_{t}\right)=E_{\pi }\left[R_{t}|s_{t},a_{t}\right]$$

where $Q^{\pi }\left(s_{t},a_{t}\right)$ represents the action–value function. A broadly used reinforcement-learning approach is learning the stated action–value function by means of an optimization process that has as its main objective to estimate the optimal Q-function (which, in the context of discrete action spaces, leads to an optimal policy of the agent). Other algorithms aim at directly estimating the agent optimal policy $\pi^{⋆}\left(s_{t}\right)$ by means of the parallel estimation of the Q-function (or, equivalently, the V-function [50]) and the computation of the policy gradient [51]. These methods, that follow the actor–critic paradigm and are compatible with continuous state and action spaces are known as policy-gradient methods, such as Deep Deterministic Policy Gradients (DDPGs) [51] or Trust-Region Policy Optimization (TRPO) [52]. Other recent algorithms, which compose a family of natural policy-gradient algorithms named Proximal Policy Optimization (PPO) [53], include a surrogate objective function for optimization. This family of algorithms lead to a reduction of complexity and an increase of both sample efficiency and performance. Stated increase in performance was taken into consideration for the application under study due to its high-dimensional nature of state and action spaces (refer to Section 4.3.1). Since PPO aims at directly optimizing an objective function for the policy, an instant update of the policy is controlled in order to avoid divergence and to assure optimal convergence. The strategies for constraining the policy update are diverse and a matter of research. Nevertheless, the most common strategies are based on Kullback–Leibler divergence (KL-divergence) penalty and on direct-clipping penalty (see Equation (4)).

(4) $$L_{\theta_{k}}^{\mathrm{CLIP}}=E_{\tau ∼\pi_{k}}\left[\sum_{t=0}^{T}\left[min\left(r_{t}\left(\theta \right){\hat{A}}_{t}^{\pi_{k}},clip\left(r_{t}\left(\theta \right),1-\epsilon ,1+\epsilon \right){\hat{A}}_{t}^{\pi_{k}}\right)\right]\right]$$

where $L_{\theta_{k}}^{\mathrm{CLIP}}$ represents the objective function for policy-weight update, $r_{t}\left(\theta \right)$ is the new–old policy ratio, ${\hat{A}}_{t}^{\pi_{k}}$ is the advantage function for policy update at time *t* and ϵ is the clipping constraint. In this work, due to computation simplicity and performance increase, PPO with a clipping penalty was selected to train the agent to accomplish the image-based multirotor following maneuver with continuous state and action spaces.

#### 4.3.1. Problem Formulation

**Figure 3:**
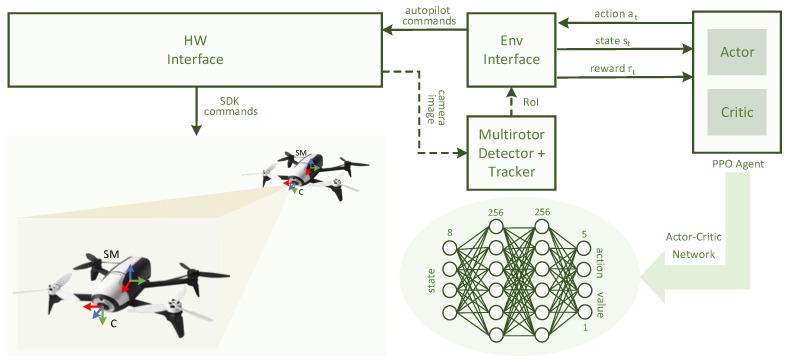
Architecture of proposed deep- and reinforcement-learning-based multirotor following system. Every involved frame of reference and architecture component is depicted. Actor–critic network, which is in charge of providing the final behavior, is structurally represented. Stabilized Multirotor (SM) frame of reference is stabilized in pitch and roll and attached to the multirotor body structure. Multirotor detector and tracker were only included in real-flight testing (dashed lines). In simulated experiments, a ground-truth Region of Interest (RoI) was generated based on projection of NC-M 3D points.

In our problem formulation, the reinforcement-learning agent perceives a continuous state $s_{t}\in S$ at time *t* (5).

(5) $$S=\{e_{cx},e_{cy},e_{area},{\dot{e}}_{cx},{\dot{e}}_{cy},{\dot{e}}_{area},\Theta_{x},\Theta_{y}\},$$

where $e_{cx}$, $e_{cy}$ and $e_{area}$ represent the normalized error of the current RoI with respect to the target RoI center position (in x and y axes) and area; ${\dot{e}}_{cx}$, ${\dot{e}}_{cy}$ and ${\dot{e}}_{area}$ represent the normalized difference of errors ($e_{cx}$, $e_{cy}$ and $e_{area}$) with respect to the previous time step and $\Theta_{x}$ and $\Theta_{y}$ are the normalized angular states of the camera gimbal in the current time step. The state is represented in the Camera (C) frame of reference (see Figure 3) and S∈ [−1, 1]. A virtual camera gimbal was added to the multirotor simulation in order to meet real platform specifications.

The reinforcement-learning agent has the ability to perform a continuous action $a_{t}\in A$ at time *t*, as represented in Equation (6).

(6) $$A=\{\theta ,\phi ,\dot{z},{\dot{\Theta }}_{x},{\dot{\Theta }}_{y}\},$$

where θ and ϕ represent the multirotor pitch and roll absolute angles, respectively; $\dot{z}$ represents multirotor altitude velocity; and ${\dot{\Theta }}_{x}$ and ${\dot{\Theta }}_{y}$ represent the camera gimbal angular difference of angles in the x and y axes, respectively. The action space is represented in the Stabilized Multirotor (SM) frame of reference for θ, ϕ and $\dot{z}$ and in the C frame of reference for ${\dot{\Theta }}_{x}$ and ${\dot{\Theta }}_{y}$ (A∈ [−1, 1]). The actions are directly forwarded to the multirotor FC as an input through SDK commands, avoiding postprocessing or filtering.

An important component in reinforcement-learning formulation is the reward function *r*, due to the high sensitivity of the current techniques to the reward-function design. Although there are some techniques that are able to deal with low-frequency and sparse-reward functions [54], most of the techniques require a more behavior-guided design. In this scenario, a well-suited reward function can decrease training times but, conversely, a weak design can introduce human bias in the final policy or even completely prevent the agent from learning a stable policy. In our presented formulation, the reward function was designed in a scheduled trend, by rewarding the main application goal higher, which keeps the target multirotor in the image plane but at the same time, by encouraging safe and smooth movements. Resulting reward function *r* is

(7) $$r=\left\{\begin{array}{cc} r_{1}=-100 & \mathrm{if}\;cx\;\mathrm{or}\;cy\\ \; & \mathrm{out}\;\mathrm{of}\;\mathrm{image}\\ r_{2}=-100 & \mathrm{if}\;area<350\;{\mathrm{px}}^{2}\\ r_{3} & \mathrm{otherwise} \end{array}\right.$$

(8) $$r_{3}=\mathrm{shaping}\left[t\right]-\mathrm{shaping}\left[t-1\right]$$

(9) $$\begin{array}{c} \mathrm{shaping}\left[t\right]=p_{center}\left[t\right]+p_{gimbal}\left[t\right]\\ +p_{area}\left[t\right]+p_{velocity}\left[t\right] \end{array}$$

(10) $$p_{center}\left[t\right]=-g_{1}\sqrt{{e_{cx}}^{2}+{e_{cy}}^{2}}$$

(11) $$p_{gimbal}\left[t\right]=-g_{2}\sqrt{{\Theta_{x}}^{2}+{\Theta_{y}}^{2}}$$

(12) $$p_{area}\left[t\right]=-g_{3}\left|e_{area}\right|$$

(13) $$p_{velocity}\left[t\right]=-g_{4}\sqrt{{{\dot{e}}_{cx}}^{2}+{{\dot{e}}_{cy}}^{2}}$$

where cx, cy and area represent the center (in x and y axes) and the area of the current RoI, respectively; $g_{1}$, $g_{2}$, $g_{3}$ and $g_{4}$ are experimentally defined constants (100, 65, 50, 30, respectively); $r_{1}$ and $r_{2}$ prevent the agent from exploring states out of a certain volume with respect to the target NC-M, based on image coordinates; and $r_{3}$ informs the agent about its instantaneous progress and helps speed up learning [55].

Furthermore, in the shaping component an explicit distinction was included in the importance of minimizing the relative image position between current and target RoI centers, the absolute position of the camera gimbal, the current and target RoI area ratio and the error velocities (each variable was weighted by a different coefficient $g_{1}$, $g_{2}$, $g_{3}$ and $g_{4}$). In this trend, the agent is encouraged to coarsely learn to minimize the position of the current RoI with respect to the target RoI, which retains the target NC-M within the camera Field of View (FOV). Subsequently, the agent is encouraged to optimize its behavior in order to keep the camera gimbal in a centered position (angles close to zero) and to finally keep a certain distance with respect to the target multirotor (RoI area directly related to the distance) as well as to decrease image velocities. Particularly, we found out that the incorporation of $\Theta_{x}$ and $\Theta_{y}$ components, weighted by $g_{2}$ coefficient, is a determinant for learning convergence. As stated, this component of the reward function encourages the agent to keep the camera gimbal in a centered position during the execution of the maneuver, diminishing the uncertainty of the solution space and making the desired behavior more explicit, that is, centering the camera gimbal provides the RL-M with more reaction time in case of sudden movements by the NC-M. Finally, the final reward was not normalized. Although $p_{center}\left[t\right]$, $p_{gimbal}\left[t\right]$, $p_{area}\left[t\right]$ and $p_{velocity}\left[t\right]$ by far exceeded the unitary value during training, the final computation of reward $r_{3}$, which informs the agent about its instantaneous progress, did not. In the context of this application and reward-function design, final reward $r_{3}$ is in the decimal order of magnitude, with values near the unit. Thus, taking this into consideration and the algorithm involved, reward normalization was not required.

#### 4.3.2. System and Network Architecture

A versatile system architecture was designed and implemented, taking the standardization of the virtual and real contexts as the main consideration. In this scenario, most of the component interfaces were shared for both simulated and real-flight experiments. A global overview of the system architecture is depicted in Figure 3. From the side of the agent, the PPO component, which had the actor–critic network as its motion-policy representation model, was wrapped with an ROS [56] interface. This design not only increased the similarity between the virtual and real contexts but also reduced the friction of interaction with robotic components, since ROS is the most common middleware in the robotic ecosystem. Conversely, the implemented environment interface is in charge of parsing the raw information (target NC-M RoIs and angular states of the camera gimbal) in order to properly adapt it to the reinforcement-learning formulation (refer to Section 4.3.1). It is also in charge of interacting through SDK commands with the hardware interface, which can be real or simulated due to standard implementation. The RoI of the target NC-M was generated based on the projection of the multirotor ground-truth 3D points and the intrinsic camera parameters for the simulated environments and based on the multirotor detector, described in Section 4.1, for real-flight experiments. Hence, in order to send state $s_{t}$ to the PPO agent, the environment interface performs all the required processing by relying on current and target RoIs.

The actor–critic neural network is a feed-forward neural network with two hidden layers of 256 units each. The activation function of each unit of a hidden and output layers is a hyperbolic tangent (*tanh*). The input- and output-layer dimensions of the actor–critic network are based on the state, action and value dimensions (8 and 5 + 1 units), respectively (see Figure 3). The activation function of the output layer is bounded to the range of [−1,1] and is provided by a linear unit for the value, to output an estimation of the V-function, used to compute an advantage function [53]. It has to be remarked that other values of the number of hidden layers and/or hidden units have been tested. Nevertheless, the stated neural-network structure composes the minimum size in terms of hidden layers and units per layer, which allows for learning stability in the conditions described in this work. For instance, network models with 128 units and two hidden layers did not provide proper results.

## 5. Experiments

As supplementary material, the video of the experiments can be found in https://vimeo.com/352940150, and the stated open-source dataset in https://bitbucket.org/alejodosr/uav-dataset.

### 5.1. Experiment Setup

The environment and hardware interfaces were been implemented in c++11 and the neural networks were trained with the Tensorflow (https://www.tensorflow.org/) library. The multirotor detector was migrated to c++11 for fast-inference purposes. The agent was synchronous with a frequency of 20 Hz, the input of the environment interface was implemented as asynchronous (it is triggered by any sensory input, such as camera images and camera gimbal states). However, the output of the environment interface (i.e., the state and reward) is generated every time an action from the agent is received. Despite the fact that the system architecture was implemented to be nonspecific, as described in Section 4.3.2, there existed small differences between simulated and real experiment setups.

#### 5.1.1. Simulation

The simulated environment, designed for training the reinforcement-learning agent, was generated under the Gazebo (http://gazebosim.org) simulator and RotorS UAV simulator [57]. An AscTec Hummingbird multirotor was selected as the RL-M, due to its similarities with the real-flight platform. A generic NC-M was included in the simulation, based on which the ground-truth RoI was generated. An Nvidia GeForce GTX 1070 was used to train and test the reinforcement-learning simulated agent. A near replica of the real-flight software camera gimbal was implemented in simulation.

#### 5.1.2. Real Flight

The Parrot Bebop 2 was chosen as the real-flight multirotor platform for both NC-M and follower RL-M due to its frictionless integration, stability, size and manoeuvrability (see Figure 1). The motion-control policy and multirotor detector were successfully tested on an average CPU and GPU (at least 1 GB of video memory). The Parrot Bebop 2 camera gimbal has a resolution of 856 × 480, a horizontal FOV of 1.3463 rad, a maximum camera gimbal velocity of 0.22 rad/s and maximum camera gimbal angles of 0.87 and 0.61 rad in the x and y axes, respectively. Although the reinforcement-learning agent was exactly trained as stated in Section 4.3.1, that is, controlled in attitude for x and y axes and altitude velocity for z axis, it was slightly different for the real-flight platform. Parrot Bebop 2 does not allow for pure attitude control, so it was controlled in velocity (in the three axes of space). The adaptation for the commanded actions is

(14) $$v_{x}=k\theta$$

(15) $$v_{y}=k\phi ,$$

where *k* was experimentally set and represents the adaptation coefficient (0.23 m rad${}^{-1}$ s${}^{-1}$); $v_{x}$ and $v_{y}$ represent velocity in the x and y axes, respectively; and θ and ϕ are commanded pitch and roll, respectively. This adaptation is only valid for stationary values of the pitch and roll angles and contributes to the differences between the simulated and real multirotor platform.

### 5.2. Training Methodology

#### 5.2.1. Multirotor Detector

**Figure 4:**
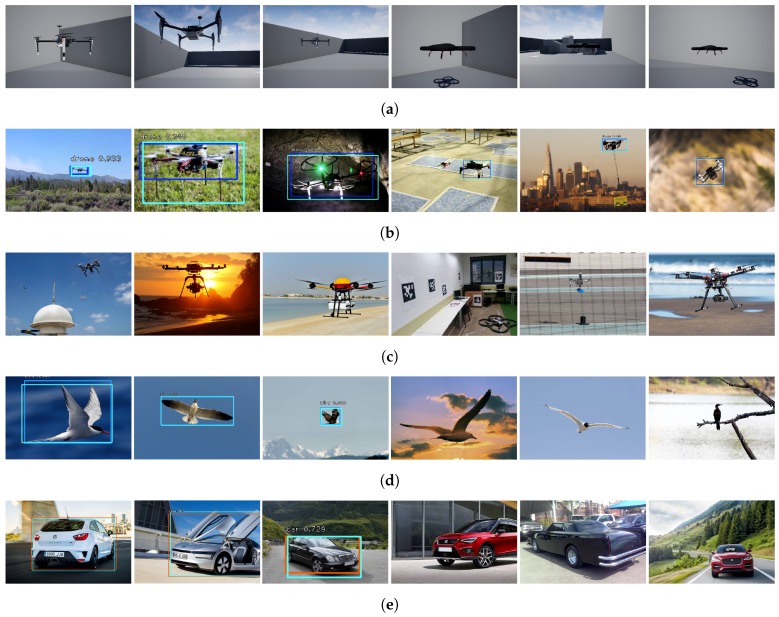
Samples from multirotor-detector results (ground-truth Region of Interest (RoI), pale blue; detected RoI, dark blue). (**a**) Training samples from synthetic dataset. (**b**) Validation samples from multirotor dataset (true positive). (**c**) Validation samples from multirotor dataset (false negative). (**d**) Validation samples from birds dataset (true positive and false negative). (**e**) Validation samples from cars dataset (true positive and false negative).

The multirotor detector was trained with standard supervised-learning techniques by means of synthetic images. These synthetic images were generated under the photorealistic Unreal (https://www.unrealengine.com) game engine and also released as an open-source dataset (see Supplementary Material). For our approach to be successful with our synthetic dataset, the detector network was pretrained on the COCO dataset [58] and fine-tuned with our syntethic dataset (new synthetic multirotor class not available in COCO dataset). Our dataset was mainly composed of two synthetic multirotor models (Parrot ArDrone and DJI Matrice 100) and a small subset of birds and cars extracted from the COCO dataset. We found that, with the addition of these two extra classes, we were able to decrease the total number of false positives. A small multirotor-validation dataset composed of real images was also annotated in order to select the most appropriate network and to provide training and validation results. The training and validation datasets were composed of approximately 8000 and 300 samples, respectively. A reduced set of samples from the training and validation datasets is depicted in Figure 4.

The whole network was trained with the Adam optimizer, with a minibatch size of 1 and the remaining hyperparameters as in Reference [47]. We fine-tuned our network for six epochs (10,000 update steps each with frozen backbone weights) and selected the best one in terms of mean Average Precision (AP) performance. In Table 1, AP is shown for every epoch of training and validation and taking into consideration in a separate trend both the full dataset and the part of the dataset corresponding to the multirotor class. Additionally, a special case was included, where the network was not yet trained with synthetic images (epoch 0). In this special scenario, since the multirotor class was not present in the COCO dataset, the airplane class was selected instead (stated class has been the closest in terms of AP, among others). The average inference time of the network has been 100 ms (Nvidia GeForce GTX 950M). Nevertheless, in order to assure a RoI in every frame during the whole experiment, as explained in previous sections, a state-of-the-art tracker was also integrated. The multirotor detector was periodically executed during the experiment in order to refine the tracker RoI. Backbone weight freezing with final subnetwork fine tuning were crucial for the detector to generalize for real-domain multirotor images based on pure synthetic multirotor-training images. Complete model retraining did not provide competent results for the application under study. Hence, the backbone extracted a high-dimensional feature map that was rich enough to extract an encoding of the input image, which was latterly classified by the final subnetworks to decide the corresponding class.

#### 5.2.2. Motion-Control Policy

The reinforcement-learning problem was treated as episodic, that is, once the agent reached an absorbing state, the episode reset and both multirotors were respawned to a uniformly randomized position within the RL-M FOV boundaries. The states where the agent perceived $r_{1}$ or $r_{2}$ as its instantaneous reward were considered absorbing states (refer to Equation (7)). The time length of an episode is limited to 1000 time steps (considering a time step of 0.05 s, as previously stated). The NC-M RoI was synthetically generated for training the reinforcement-learning agent by means of the 3D ground-truth position of the NC-M and the simulated camera gimbal intrinsic parameters. However, it showed similar performance when tested with the RoI generated by our multirotor detector and tracker. The NC-M was also completely static during the whole training process. This fact did not deteriorate the final performance since the distribution of states and actions experimented with either static or moving NC-M was equivalent and the actor–critic network did not take temporal structure into consideration (feed-forward neural network).

**Figure 5:**
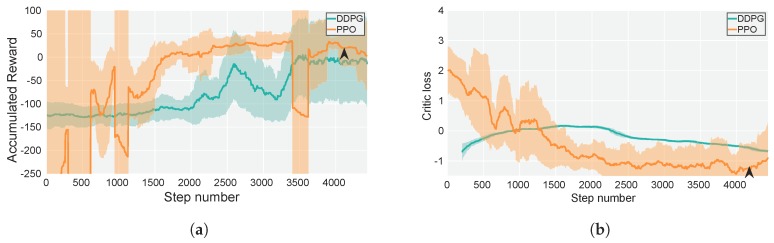
Moving average and standard deviation over 50 update steps of the training. (**a**) Accumulated Reward and (**b**) Critic loss function (logarithmic representation). The network corresponding to update step 4220 provided the best performance.

As shown in Figure 5, in order to provide a baseline for training, DDPG has been included in the study. Hence, both DDPG and PPO algorithms has been trained in the same conditions. Nevertheless, DDPG has not provided adequate results in terms of practical performance, even when the average of the accumulated reward seems to reach similar values with respect to PPO. Considering these results, only PPO has been selected for further experimentation (refer to Section 6 for more in-depth details). Both DDPG and PPO actor-critic networks have been trained with Adam optimizer, with a learning rate of $3\times 10^{-4}$ and a minibatch size of 64. DDPG has been trained with a soft target update rate τ of 0.001 [51]. PPO has been trained with a policy clipping value of 0.1 [53]. The agent has been trained over 4432 update steps (with trajectories of 256 experiences) for a total time of approximately 48 h and 16 h for DDPG and PPO, respectively. Each weights update step incurs in 8 epochs with the stated minibatch size. In the case of PPO, which has been the only algorithm to provide the full desired behavior, several networks have been tested from update step 3000 to update step 4300, being able to select actor-critic network of update step 4220, which has shown the best results in terms of performance for the scenarios presented in this work. Stated performance was measured with a small subset of 10 testing trials for every network pre-selected and utilizing metrics shown in Table 2. After performance-based pre-selection, the network of episode 4220 has been exhaustively tested for both simulated and real-flights.

### 5.3. Simulated and Real-Flight Experiments

**Figure 6:**
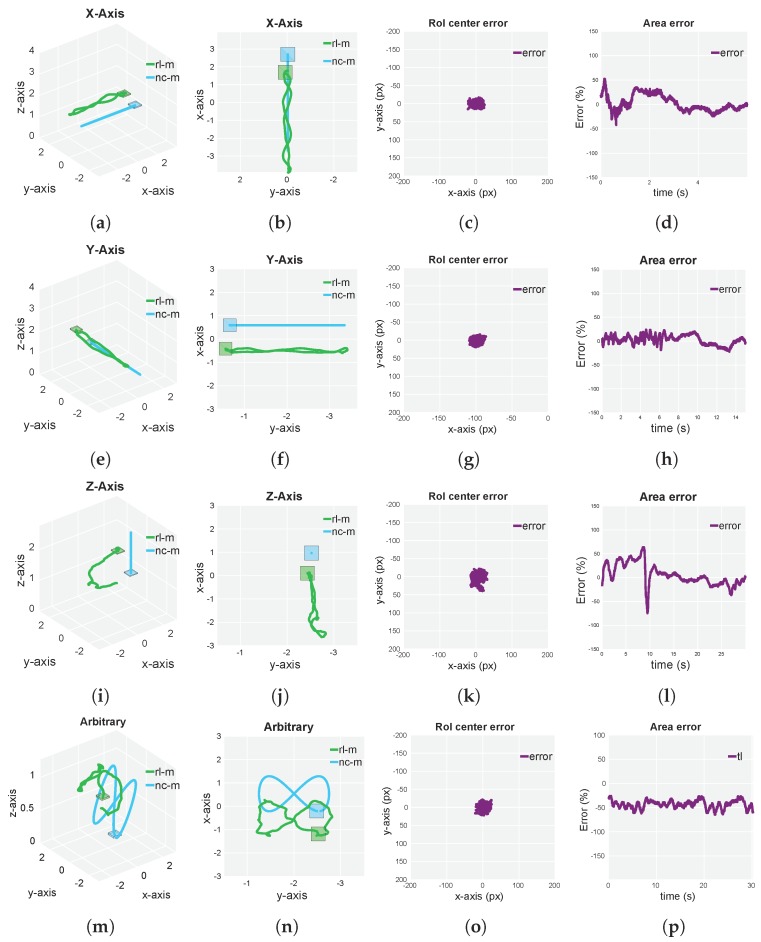
Simulation results corresponding to four different scenarios for our multirotor following approach. (**a**,**b**,**e**,**f**) Rectilinear periodic trajectory of the Non-Cooperative Multirotor (NC-M) along the x and y axes with a maximum velocity of 1.3 m/s (*X-Axis* and *Y-Axis*, respectively). (**i**,**j**) Rectilinear periodic trajectory of the NC-M along z axis with a maximum velocity of 0.5 m/s (*Z-Axis*). (**m**,**n**) Arbitrary non-periodic trajectory of the NC-M with a maximum velocity of 0.47 m/s (*Arbitrary*). (**c**,**d**,**g**,**h**,**k**,**l**,**o**,**p**) Current-target RoI center and area error corresponding to each of the four stated experiments. Multirotor trajectories have been generated by a groundtruth source.

**Figure 7:**
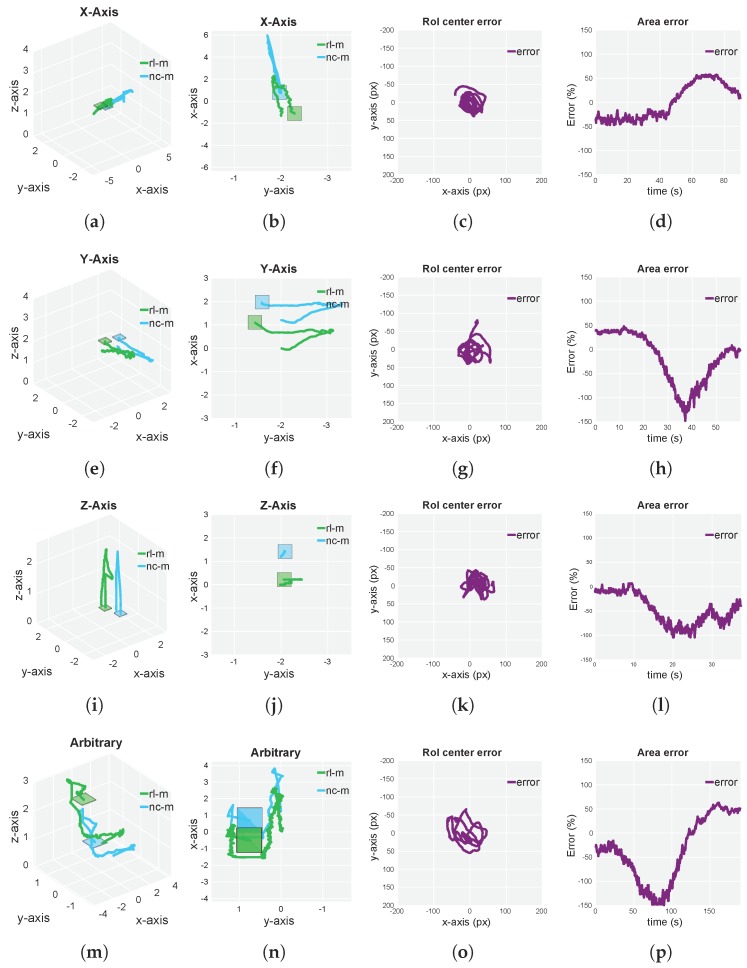
Real-flight results corresponding to four different scenarios for our multirotor following approach. (**a**,**b**,**e**,**f**,**i**,**j**) Rectilinear periodic trajectory of Noncooperative Multirotor (NC-M) along x, y and z axes with a maximum velocity of 0.3 m/s (*X-Axis*, *Y-Axis* and *Z-Axis*, respectively). (**m**,**n**) Arbitrary nonperiodic trajectory of NC-M with maximum velocity of 0.3 m/s (*Arbitrary*). (**c**,**d**,**g**,**h**,**k**,**l**,**o**,**p**) Current-target RoI center and area error corresponding to each of four stated experiments. Multirotor trajectories have been generated by an Extended Kalman Filter (EKF) that utilizes optical-flow velocities and measured accelerations as only input source.

A representative set of experiments that were able to illustrate the complete performance of our approach for both simulated and real flights were designed. Furthermore, with the aim of testing the performance of our approach in every axis of space, a separate *X-axis*, *Y-axis* and *Z-axis* experiment was carried out. In order to provide complete results about the final behavior and to bring up possible coupling interferences between axes, we performed an arbitrary experiment (refer to Figure 6 and Figure 7). Every experiment was carried out under maximum velocity conditions (that allowed RL-M to keep track of the NC-M), which were extracted from experimentation. For every performed experiment, 2D and 3D ground-truth trajectories, as well as current-target RoI center error (in pixels) and current-target RoI area error are depicted. For a complete overview of the experiment, refer to the Supplementary Material section.

## 6. Discussion

The considerable testing and validation of the presented multirotor following technique describes a proper pipeline to utilize synthetic data in the context of a challenging real-world and image-based application. It was tested in both simulated and real-flight environments in order to demonstrate the approach and to compare the differences between scenarios. On the one hand, the multirotor detector showed outstanding performance with untrained real multirotor images. It sufficiently generalized not only for the multirotor used in this work but for a wide variety of multirotors of the validation dataset (see Table 1). Epoch 0 of training showed inadequate network performance, while the following epochs showed an abrupt growth of the AP for both partial and full datasets. Results validated our technique and confirmed the benefits of the inclusion of our synthetic dataset. Additionally, this trend enables numerous applications based on multirotor detection and drafts a training pipeline for unavailable annotated datasets, such as the case of this study.

On the other hand, the multirotor motion-control policy, which was only trained in a simulated environment with NC-M in random and static positions, sufficiently generalized to perform in simulated scenarios where the NC-M was dynamically moving, as well as in real-flight scenarios with similar conditions. To the best of the authors’ knowledge, it was the first time that the camera gimbal motion was jointly controlled with multirotor motion based on image cues. Additionally, an approach where complex and image-based behavior was trained in a synthetic environment and used in the real world was validated.

The simulated and real-world environments were similar but not perfect, such as the FC response or the multirotor dynamics. Indeed, we found a decrease in performance of the approach in real-flight experiments compared with the simulated scenarios (refer to maximum velocities in Figure 6 and Figure 7). This can be due to differences between real and simulated multirotor dynamics, FC response and the camera gimbal model. Nevertheless, even with a decrease in performance, the transition of the approach from simulation to real world was direct, which was the overall aim of this work, taking into consideration the stated adaptation from Section 5.1.2.

Even though natural policy methods show outstanding data efficiency when compared with previous methods, such as the DDPG method, there are still sample inefficiencies that are a matter of community research (4432 update steps over 16 h). However, for the PPO method, both accumulated reward performance substantially improved after a few hours of training (see Figure 5). DDPG managed to improve its performance in terms of accumulated reward but, compared to the PPO method, it resulted in higher variance, a lower average of accumulated reward and longer training times (approximately 48 h). It also did not provide practical results (the variance of the agent actions was very low and usage of the camera gimbal was not present).

Regarding Figure 6, our approach showed outstanding performance in all experiment scenarios. The multirotor was able to keep track of the NC-M during the whole set of tests, being able to follow the target at a maximum velocity of 1.3 m/s for the *X-Axis* and *Y-Axis* experiments. As seen in Figure 7, the agent was able to show a similar behavior compared to the simulated environments, following the NC-M at a maximum velocity of 0.3 m/s for the arbitrary experiment. Due to differences in the simulated and real-flight autopilot capabilities, tracking the NC-M for *Z-Axis* experiment was best achieved through camera gimbal actions for simulation and through RL-M altitude velocity actions for the real-flight experiments (although keeping the NC-M within the camera FOV in both experiments).

In Table 2, we can see that both the simulated and real-flight results were in a similar order of magnitude and showed suitable behavior (the RoI was being centered during the whole experiment scenario and, in a second order of priority, the area was being compensated). The processing overhead of both multirotor detector and motion-control policy was reduced and could be executed in an average CPU (motion-control policy) and GPU (multirotor detector).

## 7. Conclusions and Future Work

In this paper, the vision-based following of a noncooperative multirotor was presented. The approach was mainly based on synthetic data and utilized deep- and reinforcement-learning techniques for the multirotor detector and motion-control policy, respectively. The multirotor detector was trained with synthetic images generated by a commercial game engine and was able to generalize to a wide range of real-world multirotors and environments. The camera gimbal and multirotor motion control were jointly treated within a motion-control policy, fully learned in a simulated multirotor environment. The complete approach was tested in both simulated and real-flight environments providing proper results, even in the presence of virtual-to-real differences in the multirotor and camera-gimbal models.

This study could be extended by improving the performance of both the multirotor detector and the motion-control policy. On the one hand, to improve the multirotor detector, the synthetic dataset could be extended and other network structures could be tested (e.g., MobileNet-SSD and YOLO). On the other hand, in order to improve the motion-control policy, both the simulated multirotor and the camera gimbal could be more realistically modeled and end-to-end fully convolutional network structure could be included as a future line of research.

## Figures and Tables

**Table 1:** Average Precision (AP) was computed for a minimum Intersection over Union (IoU) of [0.5:0.05:0.95], 0.5 and 0.75 (AP, AP${}_{50}$ and AP${}_{75}$, respectively). Network of epoch 0 corresponds to a special case (0${}^{*}$), where network was not yet trained with synthetic images (only with real images of nonmultirotor classes from COCO - Common Objects in Context - dataset) and was listed in order to make more salient the effect of our synthetic dataset. Network of epoch 4 (bold) was selected for experimentation due to its capability of generalization.

		Training			Validation			Training			Validation	
		(only NC-M)			(only NC-M)			(full)			(full)	
**Epoch**	**AP**	**AP** ${}_{50}$	**AP** ${}_{75}$	**AP**	**AP** ${}_{50}$	**AP** ${}_{75}$	**AP**	**AP** ${}_{50}$	**AP** ${}_{75}$	**AP**	**AP** ${}_{50}$	**AP** ${}_{75}$
0${}^{*}$	0.73	0.98	0.54	0.24	0.35	0.30	0.30	0.58	0.54	0.59	0.73	0.69
1	0.91	0.99	0.99	0.25	0.35	0.31	0.69	0.83	0.76	0.73	0.96	0.85
2	0.92	0.99	0.99	0.63	0.93	0.70	0.76	0.88	0.83	0.75	0.96	0.87
3	0.94	0.99	0.99	0.61	0.92	0.70	0.76	0.88	0.83	0.73	0.95	0.85
4	**0.93**	**0.99**	**0.99**	**0.64**	**0.93**	**0.75**	**0.85**	**0.95**	**0.92**	**0.75**	**0.97**	**0.89**
5	0.95	0.99	0.99	0.61	0.89	0.69	0.95	0.99	0.98	0.74	0.95	0.87
6	0.95	0.99	0.99	0.60	0.89	0.68	0.95	0.98	0.98	0.73	0.96	0.88

**Table 2:** RoI center and area error statistics for the whole set of experiments (simulated and real-flight cases). Average, maximum and minimum values are provided.

	Simulation			Real Flights		
Experiment	Center error (px)			Center error (px)		
scenario	Avg	Max	Min	Avg	Max	Min
*X-axis*	5.79	24	0	15.15	44	0
*Y-axis*	5.72	27	0	22.35	80	0
*Z-axis*	7.58	39	0	20.31	66	0
*Arbitrary*	5.7	24	0	26.71	65	0
Experiment	Area error (%)			Area error (%)		
scenario	Avg	Max	Min	Avg	Max	Min
*X-axis*	13.61	52.51	0.16	33.59	57.38	0
*Y-axis*	8.59	24.10	0.16	44.02	148	0
*Z-axis*	17.41	74.51	0	47.12	104	0
*Arbitrary*	10.94	47.56	0	63.99	162.8	0.6

## Supplementary Materials

The following are available online at https://www.mdpi.com/1424-8220/19/21/4794/s1.

## Abbreviations

The following abbreviations are used in this manuscript:

UPM	Universidad Politécnica de Madrid
CSIC	Consejo Superior de Investigaciones Científicas
DOF	Degrees Of Freedom
FC	Flight Controller
RGB	Red Green Blue
UAV	Unmanned Aerial Vehicle
PID	Proportional Integral Derivative
RoI	Region of Interest
HOG	Histogram Of Gradients
LBP	Local Binary Patterns
CAD	Computer-Aided Design
CNN	Convolutional Neural Network
NC-M	Noncooperative Multirotor
RL-M	Reinforcement-Learning-based Multirotor
FPN	Feature Pyramid Network
FL	Focal Loss
DCF	Discriminative Correlation Filter
DDPG	Deep Deterministic Policy Gradients
TRPO	Trust-Region Policy Optimization
PPO	Proximal Policy Optimization
FOV	Field Of View
ROS	Robotic Operating System
COCO	Common Objects in COntext
AP	Average Precision
EKF	Extended Kalman Filter
